# Sensitivity of hematopoietic stem cells to mitochondrial dysfunction by *SdhD* gene deletion

**DOI:** 10.1038/cddis.2016.411

**Published:** 2016-12-08

**Authors:** José Antonio Bejarano-García, África Millán-Uclés, Iván V Rosado, Luís Ignacio Sánchez-Abarca, Teresa Caballero-Velázquez, María José Durán-Galván, José Antonio Pérez-Simón, José I Piruat

**Affiliations:** 1 Instituto de Biomedicina de Sevilla (IBiS), Hospital Universitario Virgen del Rocío, CSIC, Universidad de Sevilla, Seville, Spain; 2 Departamento de Hematología, Hospital Universitario Virgen del Rocío, Seville, Spain

## Abstract

It is established that hematopoietic stem cells (HSC) in the hypoxic bone marrow have adapted their metabolism to oxygen-limiting conditions. This adaptation includes suppression of mitochondrial activity, induction of anerobic glycolysis, and activation of hypoxia-inducible transcription factor 1*α* (Hif1*α*)-dependent gene expression. During progression of hematopoiesis, a metabolic switch towards mitochondrial oxidative phosphorylation is observed, making this organelle essential for determining cell fate choice in bone marrow. However, given that HSC metabolism is essentially oxygen-independent, it is still unclear whether functional mitochondria are absolutely required for their survival. To assess the actual dependency of these undifferentiated cells on mitochondrial function, we have performed an analysis of the hematopoiesis in a mouse mutant, named SDHD-ESR, with inducible deletion of the mitochondrial protein-encoding *SdhD* gene. This gene encodes one of the subunits of the mitochondrial complex II (MCII). In this study, we demonstrate that, in contrast to what has been previously established, survival of HSC, and also myeloid and B-lymphoid progenitors, depends on proper mitochondrial activity. In addition, gene expression analysis of these hematopoietic lineages in SDHD-ESR mutants calls into question the proposed activation of Hif1*α* in response to MCII dysfunction.

The bone marrow (BM) is a hypoxic niche containing a unique vascular system with low oxygen tension.^1, 2, 3, 4^ Therefore, it is well established that hematopoietic stem cells (HSCs) have adapted their metabolism to such oxygen-limiting conditions, a situation termed to as hypoxia.^5, 6^ This adaptation includes suppression of mitochondrial activity and enhancement of anerobic glycolysis,^7, 8, 9^ both processes requiring induction of a set of genes mostly through the activation of the hypoxia-inducible transcription factor 1*α* (Hif1*α*).^7, 10, 11, 12^ During the process of hematopoiesis, a metabolic shift takes place leading to suppression of anerobic metabolism, increase in mitochondrial content, and induction of oxidative phosphorylation. These changes are meant to satisfy the bioenergetic demands in each cell stage, from quiescent HSC to proliferative hematopoietic progenitor cells (HPCs), and from these to terminally differentiated mature cells.^13, 14^

Therefore, given that HSC metabolism is essentially oxygen-independent, it is often assumed that mitochondrial dysfunction affects mature cells more severely than undifferentiated cells. However, in addition to its function as powerhouse of the cell, mitochondria has also crucial roles in metabolism regulation, biosynthesis of molecules, generation of reactive oxygen species, calcium homeostasis, and other processes.^15, 16^ Thus, even in the hypoxic BM, mitochondria have been proposed to be one of the determinants of hematopoietic stem cell fate choice and homeostasis,^17, 18^ whose disruption may have severe consequences for the organism.^13, 19, 20, 21^ In fact, some hematological disorders, particularly myeloid neoplasias of clonal origin, are associated to mitochondrial dysfunction. Among these are acute myeloid leukemia (AML) and myelodysplastic syndrome (MDS), in which the original malignant transformation seems to occur at the HSC compartment. Paradigmatic examples are cases of AML and MDS caused by mutations in the mitochondrial isocitrate dehydrogenase (IDH) enzyme of the Krebs cycle.^22, 23, 24^ More rarely, mutations in another related enzyme, succinate dehydrogenase (SDH; or mitochondrial complex II), are also related to the development of hematological malignancies.^25, 26^ Of interest, among the different pathological mechanisms proposed leading to tumorigenesis in these mutant backgrounds, some implicate the constitutive stabilization of Hif1*α* by a mechanism termed ‘pseudo-hypoxia'.^27, 28, 29^ However, the actual contribution of ‘pseudo-hypoxia' mechanism to mitochondrial dysfunction-induced oncogenic transformation is not completely established.^15, 30, 31^

In spite of these observations, it is still unclear whether functional mitochondria are absolutely required for survival of HSC or rather only needed for cell fate choice and differentiation. In this regard, some mouse models with mitochondria deficiencies display blockade of hematopoietic differentiation while not affecting the steady-state number of HSC.^17, 18, 21, 32^ In order to assess the actual dependency of these undifferentiated cells on mitochondrial function, we have performed an analysis of hematopoiesis in a CRE-LoxP-based knockout mouse mutant (SDHD-ESR mouse) with inducible deletion of the tumor suppressor *SdhD* gene,^33, 34^ which encodes one of the subunits of the mitochondrial complex II (MCII). This complex has a dual role in mitochondria, first, as one of the entry sites of electrons into the mitochondrial electron transport chain, and second, as the succinate dehydrogenase (SDH) enzyme of the Krebs cycle. Deletion of *SdhD* in nervous system revealed that this gene is necessary for neuronal differentiation and maturation.^33, 35^ In this study, we demonstrate that, in contrast to what has been previously established, survival of HSC, as well as some progenitors of different lineages, depends on proper mitochondrial activity. In addition, gene expression analysis of these hematopoietic lineages in SDHD-ESR mutants calls into question the ‘pseudo-hypoxia' theory, thus strengthening the existence of alternative mechanisms for tumorigenesis upon mitochondrial dysfunction proposed by our group.^34^

## Results

### Depletion of BM leukocytes in the SDHD-ESR mouse

**Figure 1:**
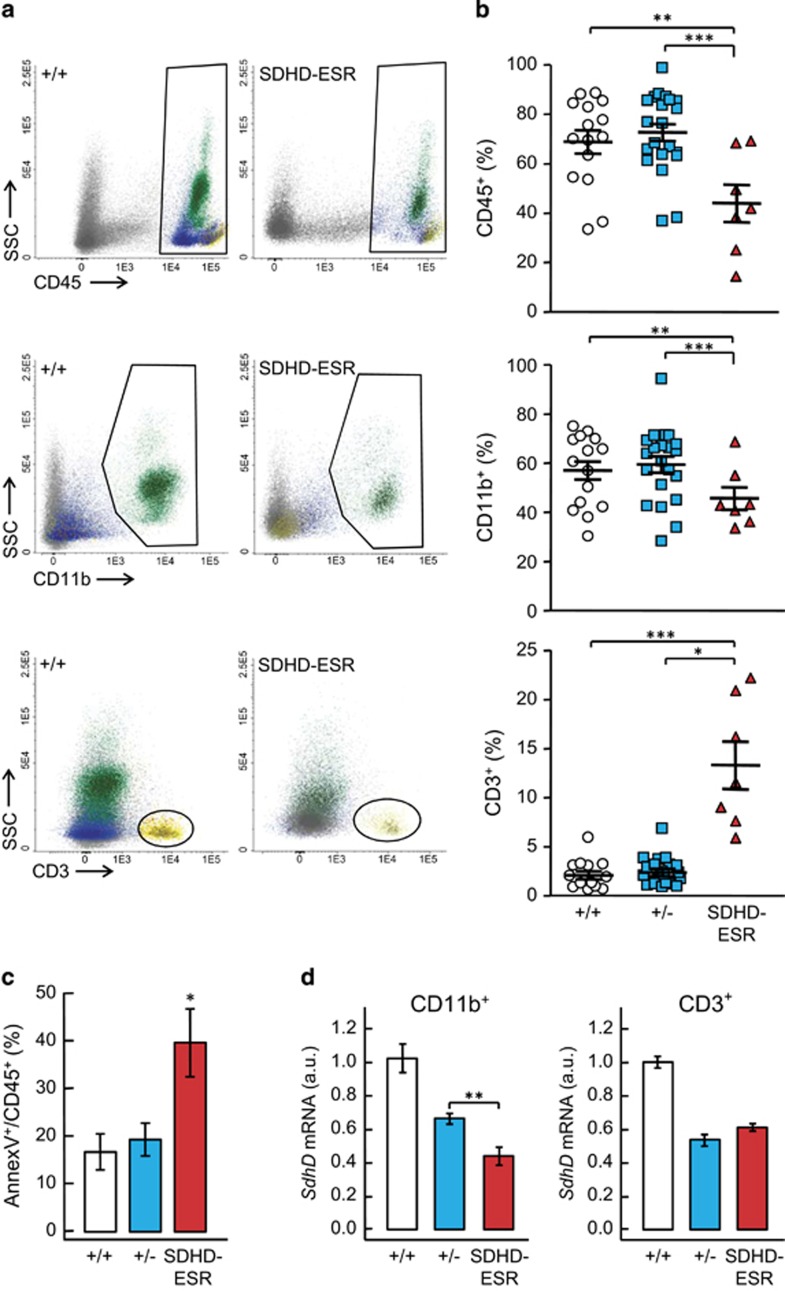
Analysis of leukocytes in bone marrow of SDHD-ESR mice. Bone marrow cell preparations were labeled with antibodies against the common leukocyte antigen, CD45; granulocyte- and monocyte-specific, CD11b antigen; and T-cell-specific CD3 antigen, for the detection by flow cytometry. (**a**) Representative dot-plots of total BM cells labeled with CD45, CD11b, and CD3 markers from *SdhD*^flox/+^ (+/+) and *SdhD*^flox/^^−^ CRE (SDHD-ESR) individuals. For simplicity, the *SdhD*^flox/^^−^ (+/−) animal is omitted as it displays the same phenotype as the +/+ control. Populations are also distinguishable by colors. SSC, side-scattered component. (**b**) Quantification of CD45^+^ leukocytes relative to the total number of events, and granulocyte/macrophage and T cells relative to total CD45^+^ cells. Each symbol represents data from a single animal. (**c**) Percentage of Annexin V^+^ events in CD45^+^ population. *N*=7–17 per genotype. (**d**) Relative *SdhD* mRNA levels in granulocytes and monocytes/macrophages (CD11b^+^) and T cells (CD3^+^). *N*=6–11 per genotype. Bars represent the mean values±S.E.M. Statistical significance: *P⩽0.05; **P⩽0.01; ***P⩽0.001. a.u., arbitrary units

In order to study the effect of *SdhD* mutation in hematopoiesis, we analyzed cell lineages in BM of SDHD-ESR mouse mutants. This model allows inducible ubiquitous CRE recombinase-mediated deletion of the *SdhD* gene by tamoxifen treatment. We have previously demonstrated that deletion of this gene decreases MCII activity in all the tissues analyzed.^36^ The systemic effect is reflected in weight loss and premature death around five weeks after tamoxifen treatment.^34^ The analysis of total leukocytes, all expressing the common leukocyte CD45 antigen, shows a decrease in the number of CD45^+^ events relative to the total number of cells gated by forward and side-scattered components (FSC/SSC) parameters two weeks after tamoxifen treatment (Figures 1a and b). The decrease in CD45^+^ cell number is accompanied by increased levels of the apoptotic marker annexin V (Figure 1c). To analyze in more detail the leukocyte lineages, we first quantified the relative numbers of granulocytes and monocytes/macrophages (CD11b^+^ cells), and T cells (CD3^+^) within the CD45^+^ population, as well as the relative level of *SdhD* messenger RNA as a manner to assess the extent of the deletion in these cell populations. In the CD11b^+^ fraction, we observed a decrease in the number of events (Figures 1a and b). Regarding T cells, we observed that this population not only did not decrease in relative number but proportionally increased upon tamoxifen treatment. Accordingly, although the level of *SdhD* transcript decreased in the pool of granulocytes and monocytes/macrophages cells, it remained unchanged in T cells (Figure 1d), indicating that either CRE-mediated recombination is not taking place in the latter cell type, or that those cells not undergoing deletion of the *SdhD* ‘floxed' allele are able to survive and rapidly repopulate the BM. In favor of the latter hypothesis is the fact that the number of viable CD45^+^ cells, is restored to wild-type levels at later time after tamoxifen treatment (Supplementary Figure 1). Indeed, the effect of this transient deletion of *SdhD* on circulating cells is minor and consists only a decrease of platelets with no effect on the rest of white and red blood cells (Supplementary Figure 2).

### *SdhD* deletion in B-cell lineage causes marked depletion of immature precursors

**Figure 2:**
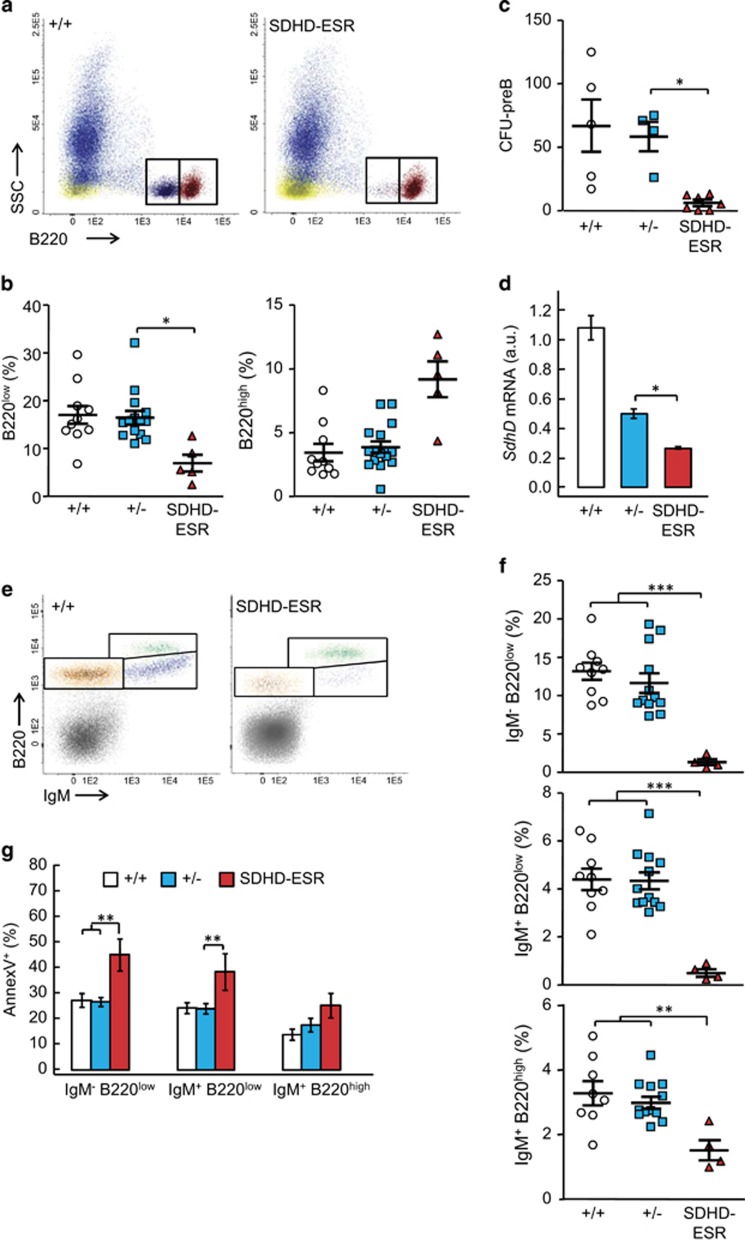
Analysis of B cells in bone marrow of SDHD-ESR mice. Bone marrow cell preparations were labeled with an antibody against the B-cell-specific antigens, B220 and IgM. (**a**) Representative flow cytometry dot-plots of CD45^+^ BM cells labeled with anti-B220 marker from +/+ and SDHD-ESR individuals. Two distinct B220^+^ populations, enclosed in rectangles, can be distinguished based on its high (B220^high^) or low (B220^low^) expression of the marker. (**b**) Quantification of B220^low^ and B220^high^ cells relative to the total number of CD45^+^ events. (**c**) Number of colony-forming units from B-cell precursors (CFU-preB) per 10^5^ cells seeded. Each dot is the average result of two different cultures from each animal. (**d**) Relative *SdhD* mRNA levels in total B220^+^ population *N*=3–11 per genotype. (**e**) Representative flow cytometry dot-plots of total BM cells expressing B220 and IgM markers from +/+ and SDHD-ESR individuals. Three distinct B220^+^ populations can be distinguished based on IgM and high (B220^high^) or low (B220^low^) B220 expression of the marker corresponding to three stages of maturation: pro-B-cell (IgM^−^ B220^low^), intermediate precursor (IgM^+^ B220^low^) and mature B-cell (IgM^+^ B220^high^). (**f**) Quantification of number of cells in each maturation stage relative to the total number of cells. (**g**) Percentage of Annexin V^+^ events in each maturation stage. *N*=4–10 per genotype. Bars represent the mean values±S.E.M. Statistical significance: *P⩽0.05; **P⩽0.01; ***P⩽0.001

Mature terminally differentiated B cells can be immunodetected in BM as B220^high^ events, whereas their immature precursors can be identified as B220^low^ events (also identifiable by their SSC^low^ CD45^low^ profile, see Figures 1 and 2). In the SDHD-ESR mutant, mature B cells relative to total CD45^+^ cells tended to increase as compared with control animals, whereas B220^low^ cell number decreased after tamoxifen treatment (Figures 2a and b). This decrease was confirmed with a functional assay in which the number of functional B-cell precursors is inferred from colony-forming units (CFUs) in culture (Figure 2c). The *SdhD* mRNA levels of the total B220^+^ population also decreased (Figure 2d). It is worth to mention that total B220^+^ population comprises both B220^high^ and B220^low^ cells, the latter being underrepresented in the mutant. Remarkably, the specific loss of B-cell precursors continued and progressed at later times (data not shown). To analyze the B220 maturation process in more detail, we identified two stages of immature B cells by adding an anti-IgM antibody. Thus, IgM^−^ B220^low^ pro-B immature stage can be distinguished from the intermediate IgM^+^ B220^low^ (Figure 2e). Both populations decreased strongly after tamoxifen treatment with respect to FSC/SSC-gated total cells. In contrast, although mature IgM^+^ B220^high^ B cells also decreased, they showed a lower sensitivity to *SdhD* deletion (Figures 2e and f). Accordingly, the analysis of viability with the apoptotic marker Annexin V indicated that although both immature B-cell populations underwent a marked increased apoptosis, a similar increase in Anexin V of the terminally differentiated B cells did not take place (Figure 2g). Together, these data demonstrate not only that B-cell lineage in the BM responds differently to the deletion of *SdhD*, but also that although the mature terminally differentiated B cells are mildly resistant to mitochondrial dysfunction, their precursors are highly sensitive.

### Thymus is severely affected by *SdhD* deletion with no specific effect on thymic T-cell precursors

**Figure 3:**
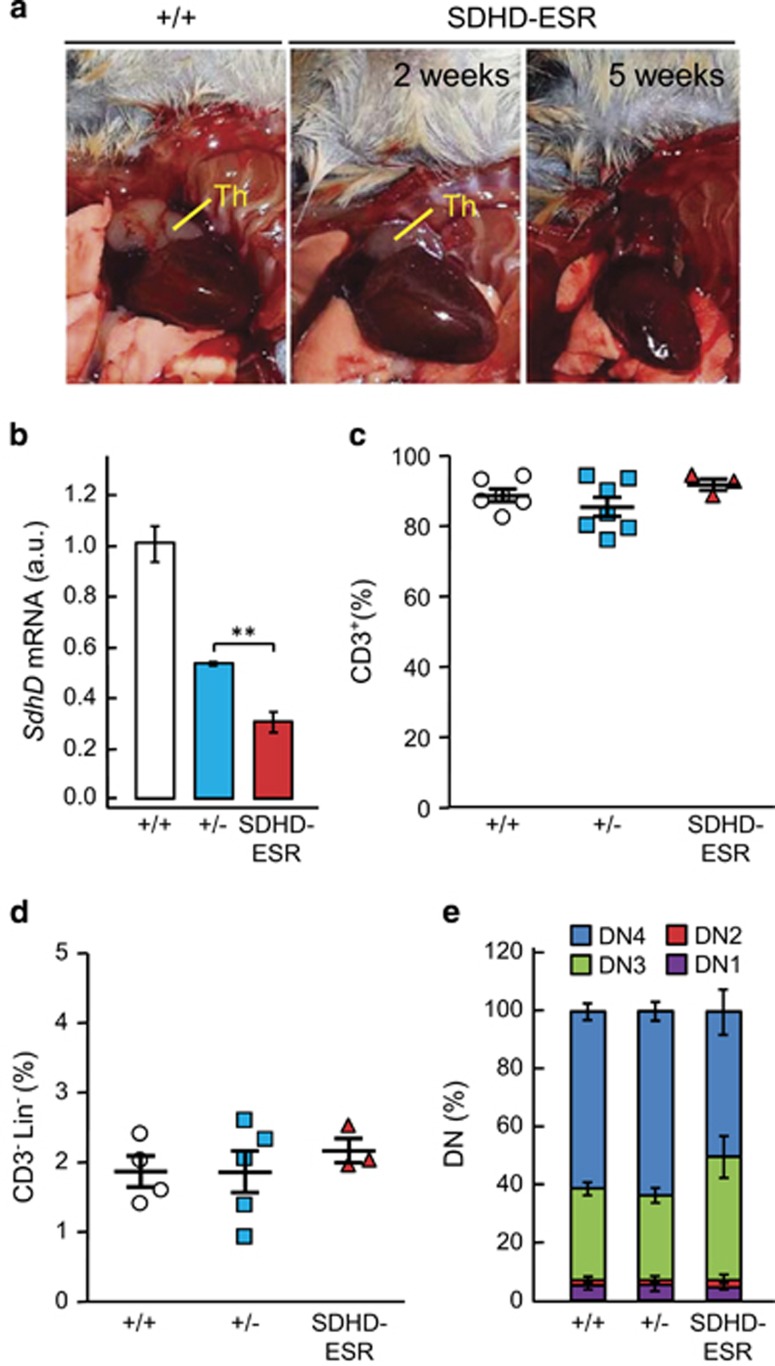
Analysis of T cells in thymus of SDHD-ESR mice at different maturation stages. (**a**) Photographs of thymi (Th) from +/+ and SDHD-ESR individuals at 2 and 5 weeks after tamoxifen treatment. Note the complete disappearance of the mutant organ after 5 weeks. (**b**) Relative *SdhD* mRNA levels in the whole organ. *N*=3–5 per genotype and group. (**c**) Quantification of T cells (CD3^+^) relative to the total number of events. (**d**) Quantification of undifferentiated thymocytes (Lin^−^ CD3^−^) relative to the total number of events. (**e**) Percentages of cells at different maturation stages, from double-negative (DN)1 to DN4, within the Lin^−^ CD3^−^ fraction. DN1 to DN4 were defined by the expression of CD44 and CD25 markers (more details in the text). *N*=3–7 per genotype. Bars represent the mean values±S.E.M. Statistical significance: **P⩽0.01

Given that loss of *SdhD* affects immature forms of B cells, we decided to analyze T-cell precursors in the thymus, the organ where maturation of T-lymphocytes takes place. We observed an atrophy of mutant thymus, which progressed towards an absolute absence of the organ as the animal deteriorates (Figure 3a). This atrophy was accompanied by the loss of *SdhD* gene (Figure 3b). The flow cytometric analysis showed no differences in the relative amount of mature (CD3^+^) T cells (Figure 3c) as well as their subpopulations (CD4^+^, CD8^+^, CD4^+^ CD8^+^, and double-negative lymphocytes; Supplementary Figure 3). To analyze T-cell precursors, we selected the lineage-marker negative (Lin^−^ CD3^−^) fraction and determined the different stages of maturation from double-negative 1 (or DN1) to DN4 by immunophenotyping CD44 and CD25 markers.^37^ Relative total number of Lin^−^ CD3^−^ precursors was not affected in the mutant (Figure 3d). Likewise, the relative subpopulations of precursors were minimally affected (Figure 3e).

### *SdhD* deletion induces loss of BM hematopoietic stem cells

**Figure 4:**
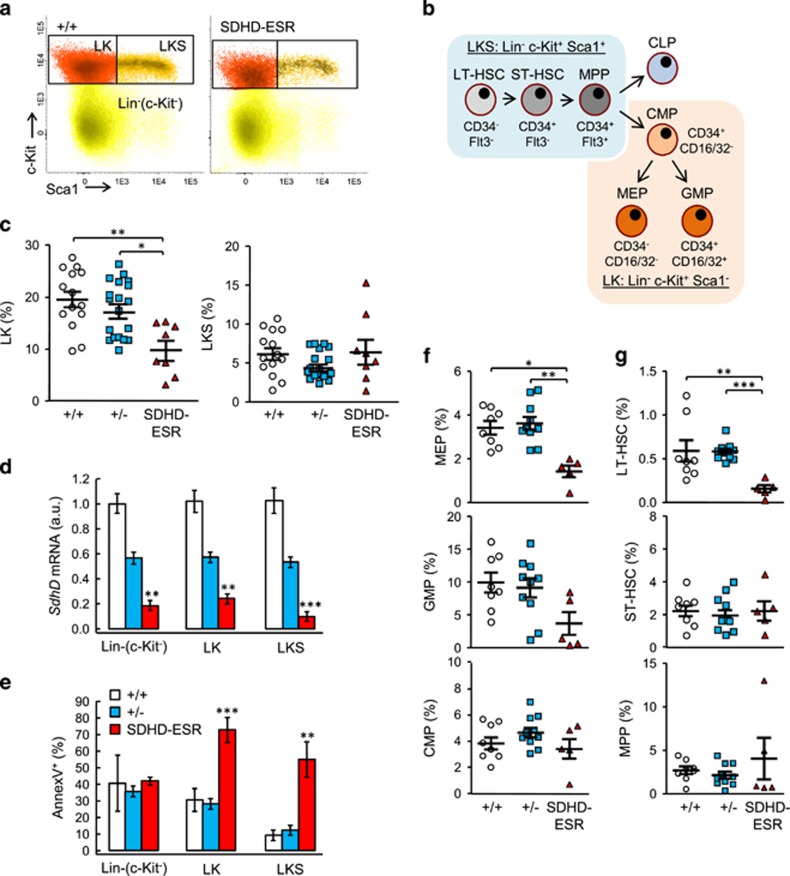
Analysis of hematopoietic stem (HSC) and progenitor (HPC) cells in bone marrow of SDHD-ESR mice. The lineage negative (Lin^−^) cell fraction was identified as described in the ‘Materials and Methods' section. The Lin^−^ cells were labeled with antibodies against c-Kit, Sca1, CD34, CD16/32, and Flt3 antigens for detection by flow cyometry. (**a**) Representative dot-plots of Lin^−^ fractions from +/+ and SDHD-ESR individuals. Rectangles indicate Lin^−^ c-Kit^+^ Sca1^−^ (LK) and Lin^−^ c-Kit^+^ Sca1^+^ (LKS) populations, also distinguishable by colors. (**b**) Diagram showing the cell types differentiated within LKS and LK populations as well as their immunophenotypes based on the expression of CD34, Flt3, and CD16/32 markers. (**c**) Quantification of LK and LKS cells relative to the total number of Lin^−^ events. (**d**) Relative *SdhD* mRNA levels in each subpopulation of the Lin^−^ fraction. *N*=5–16 per genotype and population. (**e**) Percentage of Annexin V^+^ events in each subpopulation. *N*=3–9 per genotype and population. Quantification of subpopulations contained in LK (**f**) and LKS (**g**) fractions relative to the total number of Lin^−^ events by flow cytometry with the corresponding antibodies (see the ‘Materials and Methods' section). CLP, common lymphoid progenitors; CMP, common myeloid progenitor; GMP, granulocyte/monocyte progenitor; LT-HSC, long-term HSC; MEP, megakaryocyte/erythroid progenitor; MPP, multipotent progenitors; ST-HSC, short-term HSC. Bars represent the mean values±S.E.M. Statistical significance: *P⩽0.05; **P⩽0.01; ***P⩽0.001

To study the effect of *SdhD* mutation at early stages of hematopoiesis, we analyzed in detail the population of HSC and other progenitor cells in the BM of SDHD-ESR mice. The HSC compartment can be identified by flow cytometry as lineage negative (Lin^−^) c-Kit^+^ Sca1^+^ (LKS) fraction. With the same antibodies, precursors of myeloid lineage are identified as Lin^−^ c-Kit^+^ Sca1^−^ (LK) population (Figures 4a and b). In mutant SDHD-ESR mice, the LK population undergoes a significant decrease in number relative to the total Lin^−^ fraction 2 weeks after the start of the treatment (Figure 4c). For the LKS population, such a decrease is not observed despite the fact that these cells, alike the LK and the rest of the Lin^−^ fraction, lose the expression of *SdhD* (Figure 4d). However, when apoptosis is analyzed in these same cell fractions, a strong induction is observed for both LK and LKS populations but not for the rest of Lin^−^ fraction (Figure 4e). The apparent lack of effect on the steady-state number of LKS cells can be explained by a rapid repopulation from those cells not undergoing CRE-mediated recombination. Again, at later times after tamoxifen treatment, all the parameters revert to the initial values (data not shown), thus strengthening the conclusion that those cells retaining the functional *SdhD* floxed allele could be able to replenish the corresponding cell population. We next decided to further quantify the frequency of different subpopulations within the LK and LKS compartments. Thus, by using anti-CD34 and anti-CD16/32 antibodies, the megakaryocyte/erythroid progenitors (MEP), granulocyte/macrophage progenitors (GMP), and common myeloid progenitors (CMP) fractions were identified within the LK fraction.^38^ Similarly, by using anti-CD34 and anti-Flt3 antibodies, long-term (LT) and short-term (ST) reconstituting HSC, as well as multipotent progenitors (MPP), were identified within the LKS fraction (Figure 4b).^39^ The analysis of these subpopulations shows that the decrease in LK fraction is mainly due to the loss of MEP and GMP (although statistical significance was not reached for the latter), whereas CMP remains unaltered (Figure 4f). In contrast, LT-HSC population was significantly reduced after *SdhD* deletion (Figure 4g). Therefore, contrary to the established idea that mitochondria are not required for the survival of HSC, our data indicate that most precursor cells at early stages of hematopoiesis, particularly the most immature and quiescent cells (LT-HSC), are highly sensitive to mitochondrial dysfunction.

**Figure 5:**
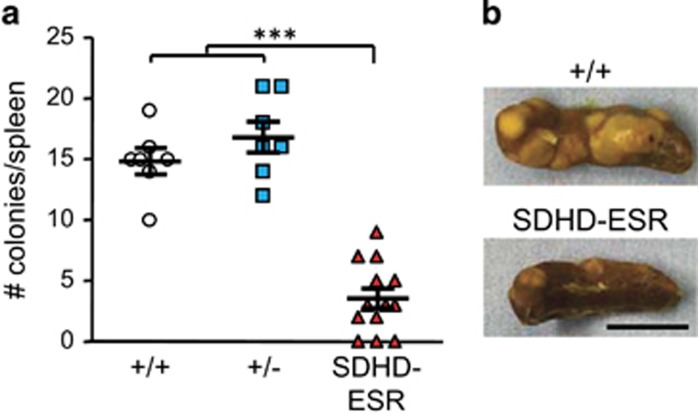
Colonies in spleen assay for detection of functional HSC. (**a**) Number of colonies in spleen per animal transplanted with bone marrow cells from two to three donors per genotype. (**b**) Representative photographs of spleens from receptors grafted with +/+ and SDHD-ESR BM cells. Bar size: 5 mm. Bars represent the mean values±S.E.M. Statistical significance: ***P⩽0.001

Nevertheless, since an effect on the number of ST-HSC and MPP was not detected, we decided to assess whether the loss of *SdhD* impaired the functionality of these cells. For this purpose, we tested the capacity of BM cells to form colonies in a clonogenic *in vivo* spleen assay (CFU in spleen at day 10; or CFU-S_10_), validated to test the multipotency and repopulating capacity of ST-HSC, and to a lesser extent, of MPP. Figure 5 shows that the number of CFU-S_10_ was drastically decreased in recipients of BM cells from SDHD-ESR mice treated with tamoxifen, thus confirming that the loss of *SdhD* gene dramatically affects the viability of HSC.

### Deletion of *SdhD* in mature granulocytes and monocytes does not exert an effect on its viability

**Figure 6:**
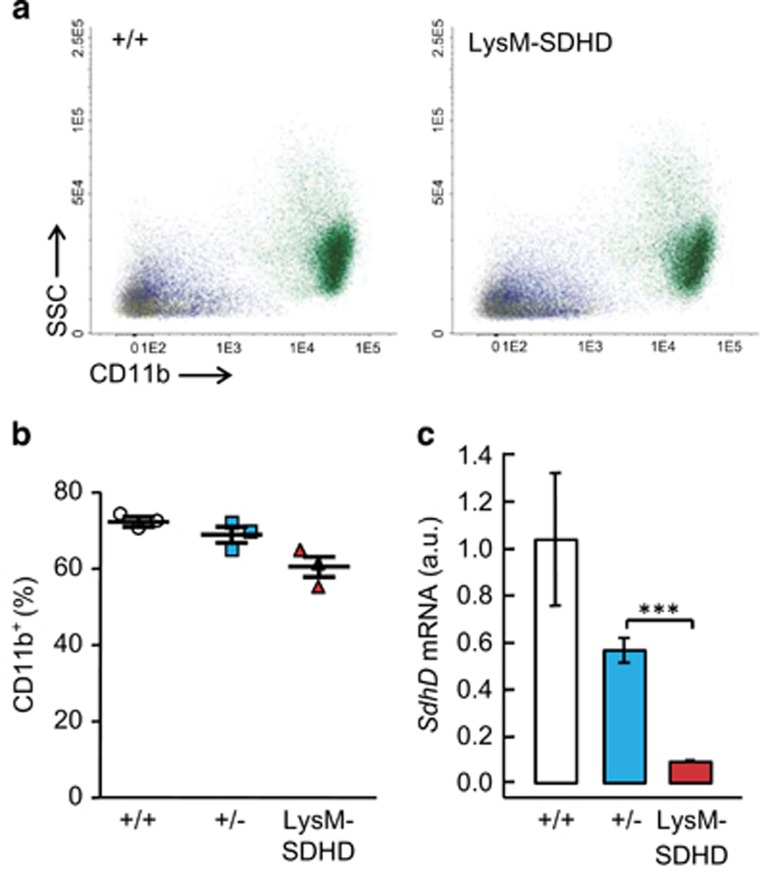
Analysis of granulocyte and monocytes in bone marrow of LysM-SDHD mice. Bone marrow cell preparations were labeled with antibodies against the common leukocyte antigen CD45, and granulocyte- and monocyte-specific CD11b; antigens for detection by flow cyometry. (**a**) Representative dot-plots of total BM cells labeled with antibodies against CD45 and CD11b markers from +/+ and SDHD-ESR individuals. SSC, side-scattered component. (**b**) Quantification of CD11b^+^ leukocytes relative to number of CD45^+^ events. (**c**) Relative *SdhD* mRNA levels in CD11b^+^ cells. *N*=3 per genotype. Bars represent the mean values±S.E.M. Statistical significance: ***P⩽0.001

To test whether deletion of *SdhD* exerted any effect on mature BM myeloid cells, a tissue-specific LysM-SDHD strain was generated by breeding the *SdhD* floxed mutant mouse with the transgenic line LysMcre, where the deletion of *SdhD* is restricted to mature forms of granulocutes and monocytes.^40^ These LysM-SDHD animals show normal appearance and survival times. The frequency of CD11b^+^ cells remained unaltered, as tested in animals at different ages from young adult (6–8 weeks) to aged (11 months) individuals (Figures 6a and b), despite the fact that *SdhD* mRNA levels were strongly decreased in this population (Figure 6c).

### Lack of pseudo-hypoxic response activation by loss of *SdhD* in BM

**Figure 7:**
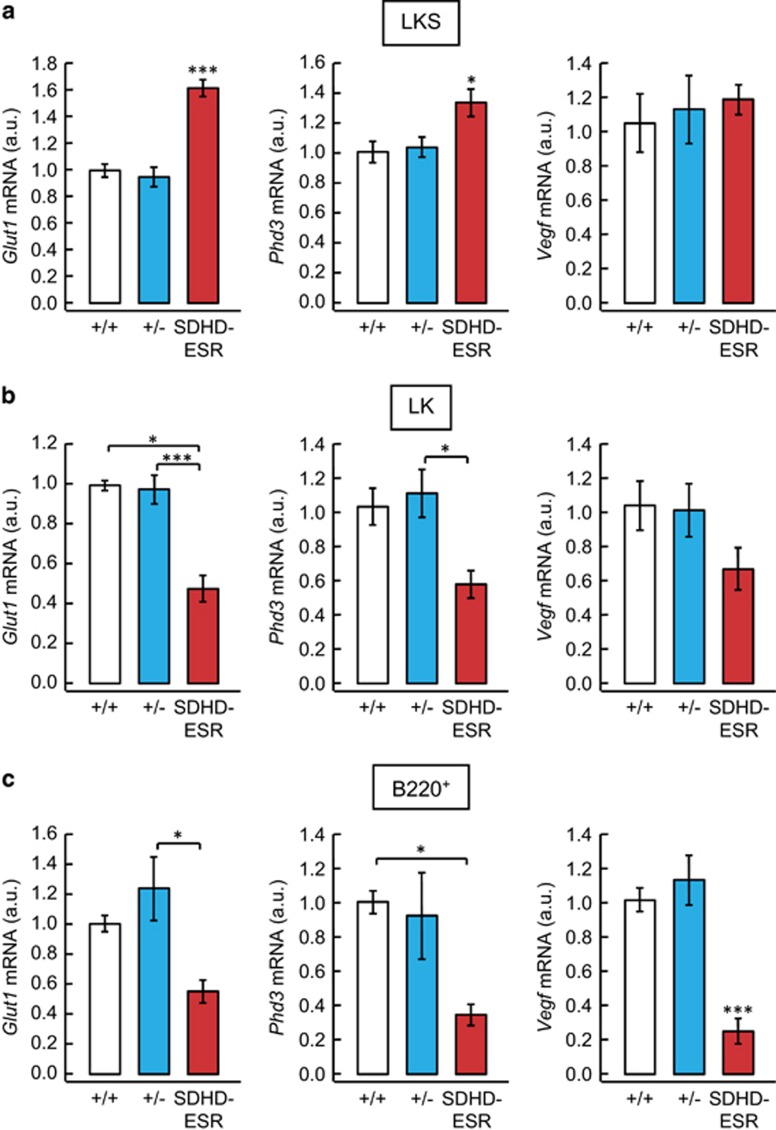
Relative mRNA levels of *Glut1, Phd3*, and *Vegf* genes in BM LKS (**a**), LK (**b**), and B220^+^ (**c**) cells of SDHD-ESR mice. *N*=3–9 per genotype. Bars represent the mean values±S.E.M. Statistical significance: *P⩽0.05; ***P⩽0.001

To shed some light on the pathways affected by *SdhD* deletion, we analyzed in LK, LKS, and B220^+^ cell populations the expression of classical HIF1*α* target genes previously studied in other SDHD-ESR tissues^34^ by determining transcript abundance. The mRNA of *Glut1* and *Phd3*, but not *Vegf,* showed increased levels only in the most undifferentiated and, supposedly most hypoxic, LKS fraction of the SDHD-ESR mutant (Figure 7a). Surprisingly, these same genes were downregulated in LK and B220^+^ cell fractions (Figures 7b and c). Although the actual biological significance of this differential response between BM cell populations remains to be investigated, our results reinforce the notion of a lack of consistent and general activation of the pseudo-hypoxia pathway in response to MCII dysfunction, thus extending what has been previously observed in other *SdhD*-deficient tissues and cell lines.^34^

## Discussion

During the last years, a direct correlation between progression along the maturation process of hematopoiesis and dependence on mitochondria has been established. Thus, based on the fact that BM is a hypoxic niche^3, 4^ and that HSC metabolism relies essentially in anaerobic glycolysis,^7, 12^ it is generally accepted that HSC are mitochondria independent.^8, 12^ It is also assumed that, as HSC progress to immature HPC and then differentiate to mature forms, they undergo a switch towards oxidative metabolism, which makes mitochondria to have an increasingly more relevant role.^9, 13, 41^ In this study, by analyzing the hematopoiesis in the mitochondria-deficient SDHD-ESR mouse mutant, we demonstrate that HSC viability and function, contrary to what has been previously established, depends on proper mitochondrial activity. Likewise, immature forms of some hematopoietic cell lines, like pre-B cells and those of myeloid lineage, also show high mitochondrial dependence for survival.

In our CRE-LoxP-based SDHD-ESR mouse, tamoxifen-induced *SdhD* deletion led to a decrease in the number of BM leukocytes at 2 weeks after the treatment, as determined by the number of common leukocyte antigen CD45^+^ cytometric events, which correlated with an activation of apoptosis. When the different populations were looked in more detail, the decrease in cell numbers was not observed for T cells, whereas granulocytes and monocytes/macrophages were mildly affected. As we were unable to detect a decrease in *SdhD* mRNA level in T cells, a very low, if any, deletion efficiency cannot be ruled out. However, given the fact that cell frequencies of most populations included in the CD45^+^ fraction, induction of annexin V, and levels of *SdhD* mRNA, are restored at later times after treatment, it is conceivable to argue that those precursors in which CRE-mediated recombination do not take place – hence retaining one functional copy of *SdhD* – are able to repopulate the BM. This would be particularly rapid for T cells, which mature outside the BM and may re-home at the hematopoietic niche.^37^ On the other hand, given that the SDHD-ESR mutants deteriorate progressively after tamoxifen administration, the reversibility of the hematological phenotype strongly supports the idea that the effect exerted by *SdhD* loss is cell autonomous. In this regard, if a systemic non-cell autonomous effect were expected to occur, the phenotype displayed by both mature and precursor (see below) cells should turn more exacerbated in time. Finally, the general conclusion suggesting that hematopoietic cells are more dependent on mitochondrial function at earlier maturation stages is strengthened by the fact that when deletion of *SdhD* is restricted to mature granulocytes and monocytes/macrophages (LysM-SDHD mouse), their steady-state frequency is practically unaffected.

A particular phenotype is observed for B cells in the BM of *SdhD* mutant mice. In this population, the negative effect of *SdhD* deletion on the number of cells is specific for immature precursors, that is, pre-B cells, as it is the induction of apoptosis. Noteworthy, the effect is sustained, and even enhanced, at later times after treatment (data not shown). The reason by which this population is particularly sensitive to mitochondrial depletion in our model is not known. Other mouse models showing particular sensitivity of B-lymphoid lineage to mitochondrial dysfunction are the so-called *Mito-mouse*, carrying large deletion of mitochondrial DNA in heteroplasmy,^17^ and a mouse model of genetic embryonic deletion of the mitochondrial ATP transport gene *Ant2*.^42^ It could also be hypothesized that, considering the persisting effect upon this lineage in the SDHD-ESR mutant, the turnover rate of pre-B cells in BM is much slower than for other populations thus impairing a rapid repopulation. Nevertheless, our model strengths the notion on that B-cell lineage is particularly sensitive to mitochondrial dysfunction. These observations are of particular interest given that, as for the myeloid lineage, defective maturation of B cells is one of the typical hallmarks of MDS.^43, 44^

At this point, it is important to remark that an effect on mature B and T cells in other hematopoiesis-related organs cannot be rule out. Spleen (not shown) and thymus are severely atrophied after loss of *SdhD*. As flow cytometric analysis of T-cell maturation in the thymus of mutant mice did not reveal differences in the relative numbers of each population, it could be hypothesized that ablation of the organ might be due to non-cell autonomous effects.

Of particular interest is the phenotype displayed by HSC and early myeloid progenitor cells. Although it is established that quiescent HSC mainly depend on glycolysis for ATP synthesis rather than on oxidative phosphorylation, it is worthwhile to mention that mitochondria also have important roles in the cell other than providing energy.^16^ For instance, supply of precursor molecules for biosynthesis of amino acids, lipids, and nucleotides is fundamental for proliferation. These molecules are synthesized *de novo* mostly using intermediates of the Krebs cycle.^11^ In addition, mitochondria have an essential role in the maintenance of the redox status of the cell and in the prevention of oxidative damage. In this regard, we^33^ and others^45, 46^ have demonstrated oxidative stress in MCII-deficient cells, which could contribute to the loss of cell viability observed in the SDHD-ESR BM. On the basis of these phenomena, mitochondria have been proposed to determine HSC quiescence and function. For instance, the aforementioned *Mito-mouse* shows altered HSC repopulating activity and differentiation towards mature forms but has no effect on HSC and HPC number and proliferation.^17^ In the same line, a mouse model carrying a proofreading-defective mitochondrial DNA polymerase displays hematopoietic defects, including anemia, lymphopenia, and myeloid lineage defects.^32^ However, accumulated mitochondrial DNA mutations in this model have little functional effect on the frequency of HSC.^21^ Moreover, a tissue-specific knockout mouse in the mitochondrial phosphatase *PTPMT1* shows defective aerobic metabolism and increased expansion of HSC.^18^ Our results contrast with these studies on that the SDHD-ESR mouse shows a clear decrease in number of LT-HSC and committed progenitors of the myeloid lineage. In addition, we report a clear functional impairment of ST-HSC and MPP. Most importantly, apoptosis is strongly induced in these populations in contrast with the rest of Lin^−^ cells despite that all of them undergo a significant loss of *SdhD* expression. Therefore, mitochondrial complex II deficiency in our SDHD-ESR mutant leads to cell death, putting into question the general idea that HSC residing in the BM do not require mitochondria for survival.

It has been proposed that loss of MCII may trigger the so-called pseudo-hypoxic drive with aberrant activation of the Hif1*α* pathway.^27, 30, 47^ Hif1*α* has proven to have a crucial role in the maintenance of the quiescent stage as well as in the stress resistance of these cells.^8, 11, 12^ Accordingly, many evidences suggest that deregulation of the Hif1*α*-mediated hypoxia response system is involved in the pathogenesis of MDS and other myeloid pathologies.^48, 49^ These observations prompted us to perform an analysis of the expression of well-established Hif1*α*-target genes in BM cell fractions with the aim of testing whether the pseudo-hypoxia response was taking place in SDHD-ESR mice. A similar study performed in other tissues and derivative cell lines by our group rendered results inconsistent with a general activation of this response.^34^ Likewise, the effect observed in BM suggests that, once again, a general activation of Hif1*α* pathway cannot unequivocally be addressed.

In summary, the characterization of hematopoiesis in our inducible *SdhD* mouse mutant has demonstrated that mitochondrial function is essential for HSC survival and maintenance. As this gene encodes for a protein involved in both, oxidative phosphorylation system and Krebs cycle, our mutant SDHD-ESR mouse allows hampering simultaneously mitochondrial bioenergetics and supply of metabolic intermediates, perhaps a condition required to reveal the actual mitochondrial dependence of HSC. This system constitutes a valid model for the study of the role of mitochondria in hematopoiesis and its possible implication in hematological malignancies.

## Materials and Methods

### Mouse strain, genotyping, husbandry, and treatment

The SDHD-ESR mouse strain, with *SdhD*^flox/−^ Cre-ERTM genotype, was generated as reported previously.^33^ The LysM-SDHD tissue-specific mouse strain was generated by breeding the *SdhD* floxed mouse strain and the LysMCre mouse strain^40^ (Jackson Laboratories, Bar Harbor, ME, USA). Littermates with *SdhD*^flox/+^ and *SdhD*^flox/−^ genotypes lacking CRE recombinase are referred to as wild-type homozygous (+/+) and heterozygous (+/−) mice, respectively. Routine genotyping was performed for the *SdhD* alleles by PCR of genomic DNA with the primers 5′-GACTAAGTGATAAACTGTCTTC-3′, 5′-GTGATATTGCTGAAGAGC-3′, and 5′-ACCCAGAACACAGACTG-3′, rendering 1.2 kb, 1 kb. and 350 bp bands in agarose gel for *Sdhd*^-^, *Sdhd*^+^, and *Sdhd*^flox^ alleles, respectively. Genotyping of Cre-ERTM transgene was performed with primers 5′-ACGGGCACTGTGTCCAG-3′ and 5′-TGTTCAGGGATCGCCAG-3′ rendering one 850 bp gel band. The mice were housed under temperature-controlled conditions (22 ºC) in a 12 h light/dark cycle, and provided with food and water *ad libitum*. Tamoxifen (Sigma-Aldrich, St. Louis, MO, USA) dissolved in corn oil was administered in doses of 150 *μ*g/g for 4 days by daily i.p. injections to 6-week-old animals.

### Flow cytometry and antibodies for cell identification

The BM cells were obtained from the tibiae and femora of mice by flushing cells with RPMI medium. The cells from thymus were obtained by mechanic disruption of the tissue. Before analysis, cell preparations were passed through a 40 *μ*m filter. Flow cytometry was performed on a BD FACS CANTO II device (BD Bioscience, Franklin Lakes, NJ, USA). The analysis was performed with the Infinicyt software (Citognos, Salamanca, Spain). For cell sorting, the samples were incubated 15 min on ice with ammonium chloride for red cell lysis and then run on BD FACSAria Fusion Cell Sorter (BD Biosciences). The antibodies used are listed in Supplementary Table 1. The lineage negative (Lin^−^) cell fraction in BM was identified as those events negative for all the following markers: B220, CD11b, CD11c, CD3, CD4, CD8, GR1, Ter119, and IL7R*α*. The markers for Lin^−^ cell fraction in thymus were: B220, CD11b, CD11c, and Ter119. Cocktails containing the corresponding antibodies, all conjugated to FITC, were used. Thymic double-negative T-cell precursors were identified as CD44^+^ CD25^−^ (DN1), CD44^+^ CD25^+^ (DN2), CD44^low^ CD25^+^ (DN3), and CD44^−^ CD25^−^ (DN4). The rest of the cell types were identified as indicated in the figure legends.

### Blood analysis

The peripheral blood was collected by heart puncture using heparinized syringes and tubes (MiniCollect 1 ml K3EDTA, Grenier Bio-One, Kremsmünster, Austria). The cells were counted on an automatic device Mythic 18 Vet (Orphée, Geneva, Plan-les-Ouates, Switzerland).

### RNA analysis

Total RNA from fresh mouse tissues and sorted BM cells was prepared using RNeasy Mini Kit (Qiagen, Hilden, Germany) or RNeasy Micro Kit (Qiagen), respectively. Reverse transcription of mRNA was performed with the QuantiTect Reverse Transcription Kit (Qiagen) and specific cDNA molecules were amplified by quantitative PCR in the presence of Power SYBR Green (Life Technologies, Carlsbad, CA, USA) with the following primers for each gene: *SdhD*, 5′-CCTGCTCTGTGGTGGACTACT-3′ and 5′-CCCATGAACGTAGTCGGTAAC-3′ *Vegf*, 5′-CGCAAGAAATCCCGGTTTAA-3′ and 5′-CAAATGCTTTCTCCGCTCTGA-3′ *Glut1*, 5′-CCAGCTGGGAATCGTCGTT-3′ and 5′-CAAGTCTGCATTGCCCATGAT-3′ *Phd3*, 5′-CAGACCGCAGGAATCCACAT-3′ and 5′-CATCGAAGTACCAGACAGTCATAGC-3′. The *Arbp* housekeeping gene was used for normalization and amplified with the primers 5′-TCCAGGCTTTGGGCATCA-3′ and 5′-CTTTATCAGCTGCACATCACTCAGA-3′.

### CFU spleen (CFU-S_10_) assay

A total of 100 000 nucleated BM cells were injected intravenously into Pep3 Boy (Jackson Laboratories) recipient mice that had been previously irradiated with 950 cGy, split in two doses with 3 h interval. Ten days after transplantation, the mice were killed, spleens were fixed in Bouin's solution (71.5% picric acid, 23.8% formaldehyde, 4.7% glacial acetic acid) for at least 12 h, and the colonies were counted.

### *In vitro* CFU assay

The number of B-cell progenitors was estimated by a CFU (CFU-PreB) *in vitro* test. For this purpose, the total BM cells were plated in duplicate using blunt-end needles at final concentration of 10^5^ cells per well in Methocult M3630 medium (Stem Cell Technologies, Vancouver, British Columbia, Canada) and incubated at 37 °C in a 5% CO_2_ atmosphere. The colonies were counted after 10 days.

### Statistical analysis

Statistical analysis was performed with the SPSS software, Chicago, IL, USA. Statistical significance of differences between groups was tested by ANOVA test for normal distribution (estimated by Shapiro–Wilk test). Variance homogeneity test will be included in the ANOVA (Levene statistics) to apply the corresponding *post hoc* test. In case of non-normal distribution, a nonparametric test (*U*-Mann–Whitney) was applied comparing groups by two.

### Ethics statement

All the experiments were performed in accordance with institutional guidelines approved by the ethics committee of the Virgen del Rocio University Hospital. The protocol was approved by the same committee according to the minute nº 02/2009.
