# Supplementary material for: Sensitivity of hematopoietic stem cells to mitochondrial dysfunction by SdhD gene deletion
Source: Cell Death Dis. 2016 Dec 8;7(12):e2516–. doi: 10.1038/cddis.2016.411 (PMC5261010; doi:10.1038/cddis.2016.411)
Supplement: Supplementary Information [file cddis2016411x1.pdf]

Supplementary Information

Sensitivity of hematopoietic stem cells to mitochondrial dysfunction by *SdhD* gene deletion

José Antonio Bejarano-García[1], África Millán-Uclés[1], Iván V. Rosado[1], L. Ignacio Sánchez-Abarca[1], Teresa Caballero-Velázquez[1, 2], María J. Durán-Galván[1], José A. Pérez-Simón*[1, 2], and José I. Piruat*[1, 2]

[1] Instituto de Biomedicina de Sevilla (IBiS), Hospital Universitario Virgen del Rocío, CSIC, Universidad de Sevilla, Seville, Spain

[2] Departamento de Hematología. Hospital Universitario Virgen del Rocío. Seville, Spain

Running title: Hematopoiesis dependency on mitochondria

*Corresponding authors:
Dr. José I. Piruat / Dr. José A. Pérez-Simón
Instituto de Biomedicina de Sevilla (IBiS)
Campus Hospital Universitario Virgen del Rocío
Avenida Manuel Siurot s/n
41013 Seville, Spain
Phone +34 955 923088
jpiruat-ibis@us.es, josea.perez.simon.sspa@juntadeandalucia.es

The Supplementary Information of this manuscript contains three supplementary figures with their legends, and one supplementary table.

Figure Legends

Figure supplementary 1. Analysis of bone marrow CD45+ cells after 2 (T2) and 5 weeks (T5) after tamoxifen treatment. (**A**) Quantification of $CD45^+$ leukocytes relative to the total number of events. Each symbol represents data from a single animal. (**B**) Percentage of Annexin V^+ events in $CD45^+$ population. N=7-17 per genotype. Bars represent the mean values ± SEM. Statistical significance: *: *P*≤0.05; **: *P*≤0.01; ***: *P*≤0.001.

Figure supplementary 2. Hematimetric analysis of circulating cells in blood of *SdhD*$^{flox/+}$ (+/+),*SdhD*$^{flox/-}$ (+/-), and *SdhD*$^{flox/-}$ CRE (SDHD-ESR) individuals after tamoxifen treatment. N=8-16 per genotype and group. Plots represent the median values in the interquartile range ± standard deviation. Statistical significance: ***: *P*≤0.001.

Figure supplementary 3. Quantification of T-cells subpopulations in thymi of SDHD-ESR mice according to the expression of CD4 and CD8 markers. Values are relative to the total number of events analyzed by flow cytometry.
