# Supplementary figures and images for: Sensitivity of hematopoietic stem cells to mitochondrial dysfunction by SdhD gene deletion
Source: Cell Death Dis. 2016 Dec 8;7(12):e2516–. doi: 10.1038/cddis.2016.411 (PMC5261010; doi:10.1038/cddis.2016.411)

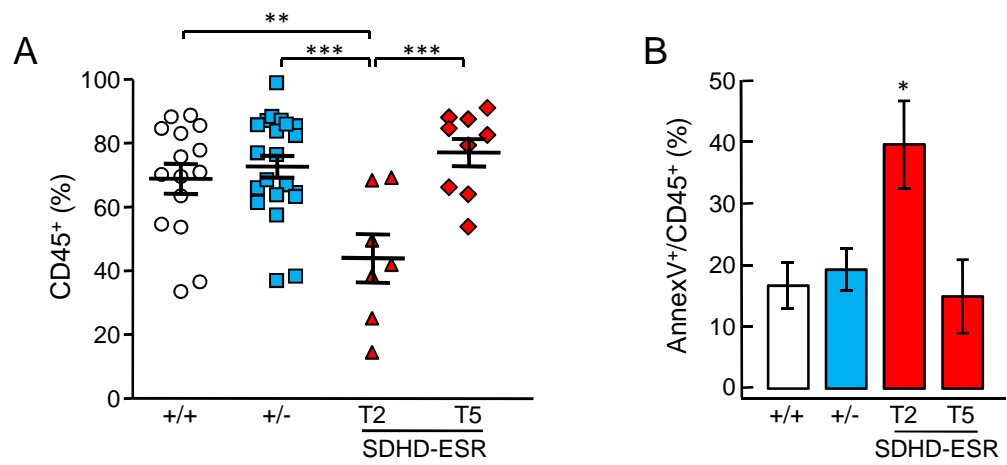

Figure supplementary 1. Bejarano-García *et al.*

Supplement: Supplementary Figure 1 [file cddis2016411x2.pdf]

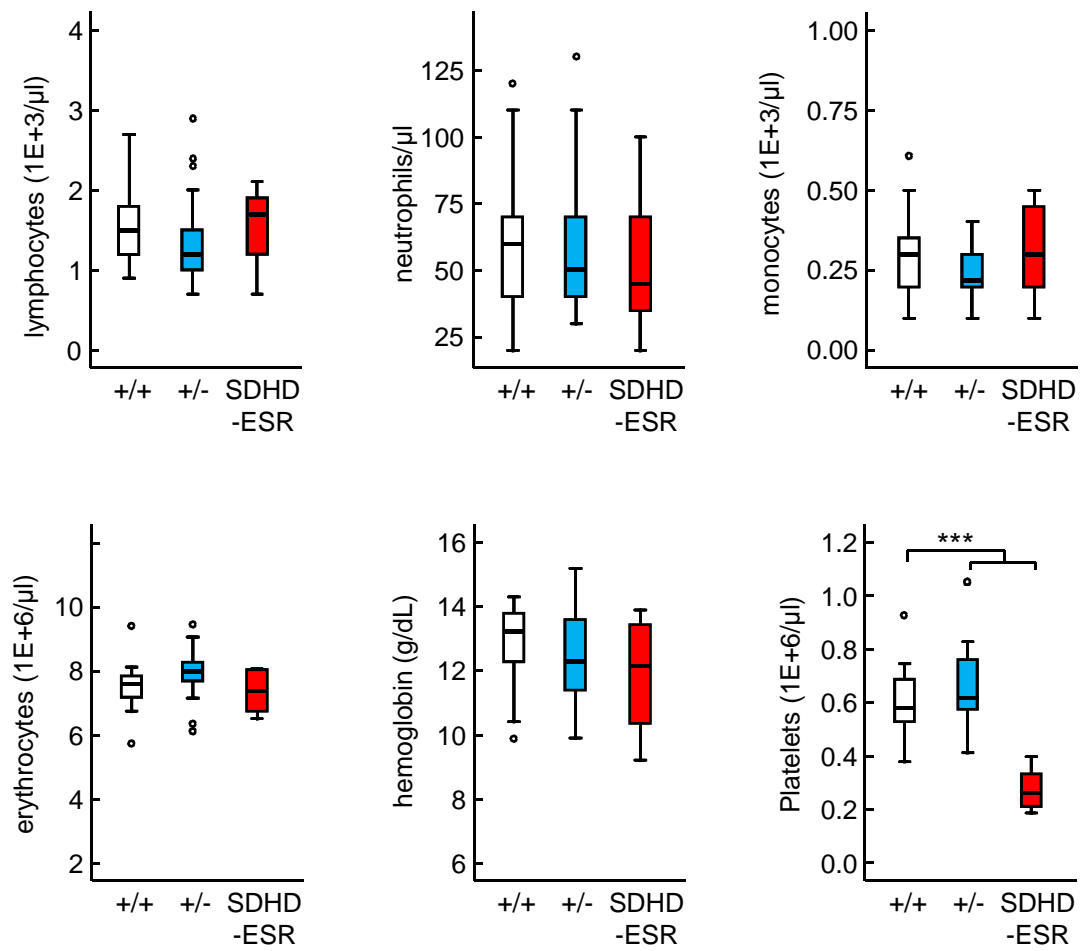

Figure supplementary 2. Bejarano-García *et al.*

Supplement: Supplementary Figure 2 [file cddis2016411x3.pdf]

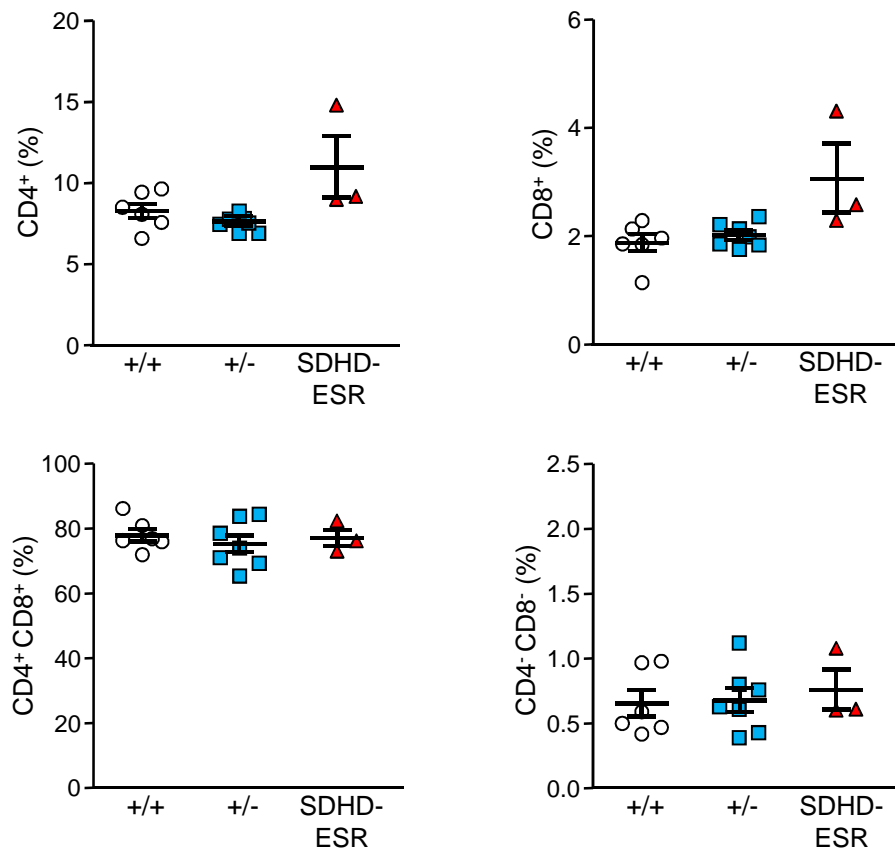

Figure supplementary 3. Bejarano-García *et al.*

Supplement: Supplementary Figure 3 [file cddis2016411x4.pdf]
