# Supplementary material for: Sensitivity of hematopoietic stem cells to mitochondrial dysfunction by SdhD gene deletion
Source: Cell Death Dis. 2016 Dec 8;7(12):e2516–. doi: 10.1038/cddis.2016.411 (PMC5261010; doi:10.1038/cddis.2016.411)
Supplement: Supplementary Table 1 [file cddis2016411x5.pdf]

Supplementary table 1. *List of antibodies used in this study*

Antibody	Fluorophore	Company	Catalog number
AnexinaV	PE	BD Pharmingen	556422
B220	FITC	Immunostep	M45RF-05MG
B220	PE	BD Pharmingen	553089
CD11b	FITC	Immunostep	M11BF-05MG
CD11b	PE	Immunostep	M11BPE-02MG
CD11c	FITC	Immunostep	M11CF-05MG
CD127	BV510	BD Biosciences	563353
CD16/32	V450	BD Biosciences	560539
CD25	PE-Cy7	BD Biosciences	552880
CD3	FITC	eBioscience	11-0031-82
CD3	PerCP Cy5	BD Pharmingen	551163
CD34	APC	eBioscience	50-0341-82
CD4	FITC	Immunostep	M4F-05MG
CD4	APC	BD Biosciences	553051
CD44	BV421	BD Biosciences	563970
CD45	APC	BD Biosciences	559864
CD71	PE	BD Biosciences	553267
CD8	FITC	BD Biosciences	553031
CD8	PE	Immunostep	M8APE-02MG
c-Kit	PerCP Cy5	BioLegend	105824
FLT3	PE	Immunostep	1399990072
GR1	FITC	Immunostep	MLY6G6CF-05MG
IgM	APC	BD Biosciences	550676
IL7R	FITC	eBioscience	11-5898-81
Sca1	PE Cy7	BD Biosciences	558162
Ter119	FITC	Immunostep	MECF-05MG
